# Does Poor Water Quality Cause Diarrheal Disease?

**DOI:** 10.4269/ajtmh.15-0689

**Published:** 2015-11-04

**Authors:** Karen Levy

**Affiliations:** Department of Environmental Health and Center for Global Safe WASH, Rollins School of Public Health, Emory University, Atlanta, Georgia

Since the time of John Snow we have known that contaminated water can lead to outbreaks of waterborne disease. This association is commonly acknowledged by both the scientific community and the lay public. But among those who study water quality and diarrheal diseases, the association is muddied by many nuanced questions.

For example, what is the relative contribution to the burden of diarrheal diseases of waterborne versus foodborne versus other transmission routes? Which pathogens are responsible for the bulk of disease? Where is the best point to intervene—at the point of contamination or at the point of ingestion?

The answers to these questions are hard to come by because of the many challenges inherent in the study of diarrheal diseases. The outcome of interest is a symptom that can be caused by a multitude of infectious agents, as well as noninfectious causes, and is difficult to accurately identify in infants, who have naturally loose stool. We often rely on self-reporting of symptoms. There are multiple routes of exposure to the panoply of pathogens that cause diarrhea—they can be ingested through water or food, or acquired by respiratory exposure. People may be exposed at home or elsewhere, leading to uncertainty in exposure characterization. Underlying health and nutritional status of the host may determine response to infections. Most studies use observational designs that offer limited potential for causal inference. Even intervention studies are often considered low quality because of their reliance on subjective outcomes and their failure to blind subjects to their treatment groups, which leads to courtesy and other biases.^1^

Observational studies examining the association between water quality and diarrhea have had mixed results. In a meta-analysis, Gundry and others^2^ found no clear relationship between point-of-use water quality and diarrhea. Subsequently Gruber and others^3^ repeated the analysis with additional studies and found a significant association between poor water quality and diarrhea for those studies that used *Escherichia coli* as an indicator of microbial water quality, but not for those that used thermotolerant coliforms. However, although the analysis identified a significant pooled association, there was heterogeneity in the individual associations, even for *E. coli*, with most studies showing positive but not significant associations.

One might conclude from this evidence that poor water quality is not as much of a cause of diarrheal diseases as we previously assumed. However, this conclusion would contradict a substantial body of evidence suggesting that *interventions* to improve water quality have consistently been associated with reduced incidence of diarrheal diseases.^2^,^4^ More likely, associations are genuine, but water quality indicators are too variable and unspecific to adequately characterize exposure to poor quality water.

Fecal indicator bacteria suffer from a “needle in the haystack” problem. Looking for specific pathogens in water is costly, time intensive, and inefficient because of the many pathogens that can cause diarrhea. Instead, we rely on surrogate indicators that are commonly present in feces. Many different indicators have been proposed and used to measure microbial water quality, but none are perfect. There is poor correlation between fecal indicator organisms and pathogens,^5^ indicators are generally not specific to humans,^6^ and measurement of indicator organisms is highly variable.^7^

However, if we understand the limits of water quality measures, we can overcome some of these limitations by increasing sample sizes^5^ and employing study designs that will help to improve the specificity of exposure and outcome measurements.

In this issue of *The American Journal of Tropical Medicine and Hygiene*, Luby and others^8^ report on a study that prospectively examined water quality in 500 households in Bangladesh, as a sub-study of the Sanitation, Hygiene Education and Water Supply in Bangladesh project. Over the course of 2 years, the researchers took water samples from households every 3 months and analyzed the relationship between water quality and diarrhea in children from the same household on a subsequent visit 3–100 days later. Luby and others found that, controlling for specific confounders (age, mother's education, wealth index, and months of surveillance), each 10-fold increase in *E. coli* contamination in drinking water was associated with a 16% increase in diarrhea in subsequent visits 3–46 days later. They saw a trend of increasing risk with higher contamination, although the prevalence ratios for the association were only statistically significant above 100 *E. coli* colony forming units/100 mL. The authors estimated the population attributable fraction (PAF) of diarrhea from contaminated water as 17%.

This study is unique in its assessment of water quality prior to diarrheal illness, and with a sufficiently large sample size to observe patterns between household water quality and diarrhea. In most of the studies in the Gruber and others review^3^ water samples were collected after or concurrently with surveys on diarrhea incidence. Only one of these studies^9^ explicitly related household water quality measurements taken on a specific day to subsequent diarrhea episodes, and this study had a smaller sample size and only two cross-sectional sampling events. Most diarrheagenic pathogens have incubations periods of > 24 hours, so the water that a researcher collects when visiting a household may not capture the disease status of the person who drinks that water. Moreover, the water collected on a particular day may not accurately reflect the quality of the water on preceding days, especially if household members change their behavior to drink higher quality water as a consequence of being ill. Also impressive was the number of water samples analyzed for this study (3,833 samples, with up to eight from each of almost 500 households).

The results of this study teach several interesting lessons. The authors point out that the measured association “likely represents a minimal estimate of the contribution of drinking water quality to diarrhea.” However, even if the PAF were doubled, contaminated water would be responsible for less than half of all diarrhea cases. In other words, the data suggest that people are getting diarrhea in many ways in addition to consumption of water in their homes. This explains in part why associations between water quality and diarrheal disease are so hard to find, especially in small studies.

In addition, those children living in households with the most consistently poor water quality suffered the worst diarrhea rates. These results suggest that priority should be placed on identifying the most heavily and consistently contaminated sites. To identify the most vulnerable individuals in a population with characteristics similar to that in this study, more frequent monitoring using cruder methods (i.e., with higher detection limits) may be a better strategy than cross-sectional sampling using highly sensitive techniques. This type of information will be useful for generalized water quality monitoring programs, which are currently under development through the World Health Organization/United Nations Children's Fund Joint Monitoring Program, as part of the proposed Sustainable Development Goals, which aim to address water quality and not just access.^10^

Going forward, improving our understanding of the relationship between water quality and diarrheal diseases will require incorporating more specificity in both exposure and response variables. Innovations to improve specificity in exploring water–diarrhea relationships may include direct testing for pathogens, high-volume sampling, more objective health endpoints, indicators that are more specific to humans and correlated with pathogens, and innovative study designs. Luby and others' study takes a step in this direction by incorporating an appropriate temporal relationship between water exposure and disease outcome, a design that should become the norm in studies of water quality and diarrhea.

## References

[1] Engel RE, Lim SS (2013). Does clean water matter? An updated meta-analysis of water supply and sanitation interventions and diarrhoeal diseases. Lancet.

[2] Gundry S, Wright J, Conroy R (2004). A systematic review of the health outcomes related to household water quality in developing countries. J Water Health.

[3] Gruber JS, Ercumen A, Colford JM (2014). Coliform bacteria as indicators of diarrheal risk in household drinking water: systematic review and meta-analysis. PLoS One.

[4] Wolf J, Pruss-Ustun A, Cumming O, Bartram J, Bonjour S, Cairncross S, Clasen T, Colford JM, Curtis V, De France J, Fewtrell L, Freeman MC, Gordon B, Hunter PR, Jeandron A, Johnston RB, Mausezahl D, Mathers C, Neira M, Higgins JP (2014). Assessing the impact of drinking water and sanitation on diarrhoeal disease in low- and middle-income settings: systematic review and meta-regression. Trop Med Int Health.

[5] Wu J, Long SC, Das D, Dorner SM (2011). Are microbial indicators and pathogens correlated? A statistical analysis of 40 years of research. J Water Health.

[6] Harwood VJ, Staley C, Badgley BD, Borges K, Korajkic A (2014). Microbial source tracking markers for detection of fecal contamination in environmental waters: relationships between pathogens and human health outcomes. FEMS Microbiol Rev.

[7] Levy K, Hubbard AE, Nelson KL, Eisenberg JN (2009). Drivers of water quality variability in northern coastal Ecuador. Environ Sci Technol.

[8] Luby SP, Halder AK, Huda TM, Unicomb L, Islam MS, Arnold BF, Johnston RB (2015). Microbiological contamination of drinking water associated with subsequent child diarrhea. Am J Trop Med Hyg.

[9] Gundry SW, Wright JA, Conroy RM, Preez MD, Genthe B, Moyo S, Mutisi C, Potgieter N (2009). Child dysentery in the Limpopo Valley: a cohort study of water, sanitation and hygiene risk factors. J Water Health.

[10] Dora C, Haines A, Balbus J, Fletcher E, Adair-Rohani H, Alabaster G, Hossain R, de Onis M, Branca F, Neira M (2015). Indicators linking health and sustainability in the post-2015 development agenda. Lancet.

